# *Rosa* spp. Extracts as a Factor That Limits the Growth of *Staphylococcus* spp. Bacteria, a Food Contaminant

**DOI:** 10.3390/molecules26154590

**Published:** 2021-07-29

**Authors:** Joanna Milala, Lidia Piekarska-Radzik, Michał Sójka, Robert Klewicki, Bożena Matysiak, Elżbieta Klewicka

**Affiliations:** 1 Institute of Food Technology and Analysis, Faculty of Biotechnology and Food Sciences, Lodz University of Technology, ul. Stefanowskiego 4/10, 90-924 Łódź, Poland; michal.sojka@p.lodz.pl (M.S.); robert.klewicki@p.lodz.pl (R.K.); 2 Institute of Fermentation Technology and Microbiology, Faculty of Biotechnology and Food Sciences, Lodz University of Technology, ul. Wólczańska 171/173, 90-924 Łódź, Poland; lidia.piekarska-radzik@dokt.p.lodz.pl; 3 The National Institute of Horticultural Research, Department of Applied Biology, 96-100 Skierniewice, Poland; bozena.matysiak@inhort.pl

**Keywords:** *Rosa* spp., antibacterial activity, *Staphylococcus* spp., phenolic

## Abstract

Due to their richness of bioactive substances, rose hips are a valuable raw material for obtaining extracts with potential antimicrobial activity. The aim of the study was to determine the antagonistic potential of whole pseudo-fruit and flesh extracts of three *Rosa* sp. varieties against *Staphylococcus* spp. bacteria isolated as food contaminants. The biological material in this study consisted of seven strains of bacteria from the genus *Staphylococcus*. Two strains—*Staphylococcus aureus* ATCC 25923 and *Staphylococcus epidermidis* DSMZ 3270—were used as reference strains. The other five strains were food-derived isolates—*S. epidermidis* A5, *S. xylosus* M5, *S. haemolyticus* M6, *S. capitis* KR6, and *S. warneri* KR2A. The material was the pseudo-fruits of *Rosa canina*, *Rosa pomifera* Karpatia, and *Rosa rugosa*. The polyphenols were extracted from the fleshy part and the whole pseudo-fruit for all rose varieties. The tested preparations differed significantly in their polyphenol composition. The sum of polyphenols ranged from 28 862 to 35 358 mg/100 g of lyophilisate. The main groups of polyphenols found in the preparations were flavanols and ellagitannins. All of the tested extracts inhibited the growth of staphylococci at a concentration of 500 mg/mL. *Rosa rugosa* fruit extract showed the strongest antimicrobial properties among the studied extracts. For all the strains, the growth inhibition had a diameter of 20.3–29.0 mm. Moreover, six out of the seven tested strains showed the highest inhibition with the use of this extract. The MIC of rose extracts was in the range of 3.125–500 mg/mL and was strictly dependent on the bacterial species, the species of the rose, and the part of the fruit from which the extract was obtained. Correlations were assessed between the main groups of polyphenols in the extracts and their inhibition of bacterial growth. In the case of pseudo-fruit extracts, the inhibitory effect on bacterial growth positively correlated with the content of ellagitannins, and this effect was observed for almost all the tested strains. The results presented herein follow the current trend of minimising the use of chemical preservatives in food; from this point of view, rose extracts are very promising.

## 1. Introduction

Bacteria belonging to the genus *Staphylococcus* are commonly found in the natural environment: in water, soil, and air. These microorganisms are an integral component of the microflora of the skin and mucous membranes of humans and warm-blooded animals. In addition, they are also found in foods such as fermented meat products, milk, and cheese [1]. *Staphylococcus aureus*, an opportunistic pathogen, is the most common cause of food poisoning in humans [2]. The methicillin-resistant strain of *S. aureus* (MRSA) was first described in 1961. Currently, more than 50% of *S. aureus* isolates derived from clinical specimens are MRSA. These bacteria are resistant not only to β-lactam antibiotics, but also to macrolides, clindamycin, quinolone, and other antimicrobial compounds [3].

The second group of staphylococci are coagulase-negative strains, which are much less frequently identified as pathogens, but are food contaminants. Some species of this genus—*S. carnosus*, *S. equorum*, *S. succinus*, and *S. xylosus*—are naturally occurring bacteria in fermented foods. These bacterial species are often a component of the starter cultures used in the production of fermented sausages. Thanks to their metabolic activity, the aromatic qualities of the product are improved and the desired texture and taste are obtained. *Staphylococcus* sp. bacteria are characterised by their ability to reduce nitrates to nitrites and further to nitrous oxide, consequently improving the colour of sausages [4].

Nevertheless, *S. epidermidis*, *S. haemolyticus*, and *S. saprophiticus* have been described as factors of opportunistic infection [4]. *S. epidermidis* is described in the literature as part of the natural microflora of the human skin. It should be kept in mind that in the case of defects in the human immune system, *S. epidermidis* can cause severe systemic infections and sepsis. Globalisation and the pace of technological development in the production of highly processed food favour the spread of these microorganisms. Additionally, the frequent use of antibiotics in treating humans and farm animals increases the number of antibiotic-resistant strains of the species *S. aureus* and other antibiotic-resistant species of *Staphylococcus* [5]. Therefore, it is extremely important to search for factors of natural origin to limit the rising numbers of *Staphylococcus* strains contaminating food and their spread.

Plant extracts obtained from various parts have a high potential for limiting the growth of microorganisms. Plants from the rose family (*Rosaceae*), particularly roses (*Rosa* sp.), have been considered to be a valuable plant material for many years due to their bioactive substances with antioxidant properties, such as flavonoids (especially catechins, proanthocyanidins, and anthocyanins), phenolic acids, vitamins (B_1_, B_2_, B_9_, C, K, and E), carotenoids, and tocopherols. These compounds are present in the petals, pseudo-fruits, and achenes [6,7]. Among these compounds, vitamin C is the most abundant. The pseudo-fruits can contain as much as 12,000 mg%, although it is usually between 840 and 3500 mg% [8,9]. The content of vitamin C depends on the variety, harvest date, post-harvest procedure, drying method, storage conditions, and storage duration [8,10]. Wild rose hips (*Rosa canina*) are an abundant source of carotenoids. There are significant amounts of β-carotene and lycopene—compounds that are precursors of vitamin A [8,11,12]. Other carotenoid pigments identified in this raw material are α-carotene, γ-carotene, lutein, zeaxanthin, rubixanthin, taraxanthin, and poly-cis lycopene A and B [10].

*Rosa* spp. are a valuable source of polyphenols [7,8,12,13,14,15,16]. The literature on the subject indicates that the content of phenolic compounds in the fruit of various species of the *Rosa* genus is estimated to be 55–104 mg/g [15,17]. These substances include hydrolysable and non-hydrolysable tannins, quercetin and kaempferol derivatives, anthocyanins, and phenolic acids (gallic, coumaric, and protocatechuic acid) [12,15,18]. Ellagic acid and ellagitannins deserve special attention due to their documented powerful anti-cancer, anti-inflammatory, and antioxidant activity [19]. Teleszko et al. [20] reported that the different varieties of rose hips they tested contained from 103.53 mg to 124.73 mg of total ellagic acid per 100 g of dry matter, depending on the variety. Ellagic acid also occurs in a bound form in hydrolysable tannins, i.e., ellagitannins such as tellimagrandin (I and II) or rugosines (A, B, D, and E) [21,22,23,24]. Apart from ellagic acid, non-hydrolysable tannins have a decisive influence on the antiradical activity of rose pseudo-fruits [20,24,25]. An analysis of phenols in wild rose identified the presence of 15 individual proanthocyanidin aglycones and 19 glycosides as major phenols [24]. The concentration of proanthocyanidin polymers present in the pseudo-fruits of three different species of the *Rosa* genus investigated by Teleszko et al. [20] ranged from 21,222 μg/g DM (dry matter) to 44,717 μg/g DM.

The flavonoid compounds present in rose tissues included the following: quercetin, isoquercitrin, rutin, and astragalin. In addition, there were significant amounts of flavonoid glycosides such as kaempferol 3-*O*-(6”-*O*-*E*-p-coumaroyl)-β-d-glucopyranoside and kaempferol 3-*O*-(6”-*O*-*Z*-p-coumaroyl)-β-d-glucopyranoside [10]. Anthocyanins, along with carotenoids, create the orange-red colour of the fruit and the pink colour of wild rose flowers. These compounds are represented by cyanidin-3-glucoside, cyanidin-3,5-diglucoside, peonidin-3-glucoside, peonidin 3,5-di-*O*-glucoside, and peonidin 3-*O*-sophoroside [12,26]. Hydrolysable tannins are known for their antioxidant activity, selective antagonistic activity, and the ability to inhibit the activity of digestive enzymes. The study by Ochir et al. [27] found that the biological activity of hydrolysable tannins is structure-dependent. Hydrolysable tannins isolated from *Rosa rugosa*—rugosin D and tellimagrandin II—show antagonistic activity against *E. coli*, *S. aureus*, *B. cereus*, and *Salmonella* spp., while they are weakly antagonistic against *Bifidobacterium breve* and *Lactobacillus salivarius* [28]. Additionally, a positive correlation was demonstrated between the content of polyphenols and the high antioxidant activity of rose extracts with high antimicrobial activity and α-amylase inhibitory activity [29].

Many studies on the antimicrobial properties of rose extracts focus on extracts obtained from rose petals; however, there are also reports on extracts obtained from other morphological parts of the plant, such as its leaves, roots, and fruits [14,30,31,32,33,34]. Tatke et al. [30] tested raw water and methanol extracts from *R. damascena* flower petals. They found an antibacterial effect of the water extract and the methanol extract against *S.aureus*, *E.coli*, *K. pneumoniae*, *K. aerogens*, *P. aeruginosa* (only methanol), *S. pyogens*, and *C. perfringens*. Moreover, the authors reported antifungal activity by methanol extract against *C. albicans*. They demonstrated that the methanol extract showed higher antioxidant and antibacterial potential. Similar results were obtained by Ulusoy et al. [31], who reported strong antibacterial properties of damask rose oil and absolute against strains of *E. coli*, *P. aeruginosa*, *B. subtilis*, *S. aureus*, *Chromobacterium violaceum*, and *Erwinia carotovora*, which are plant pathogens. *C. violaceum* turned out to be the most sensitive to both extracts of all the strains tested, while *E. coli* was the most sensitive to the essential oil. The experiments by Olech et al. [13] showed that galenic preparations from the roots, leaves, and flowers of *R. rugosa* have high antioxidant activity, as well as a high or medium ability to fight Gram-positive and Gram-negative bacteria.

Due to their richness of bioactive substances, rose hips are a valuable raw material for obtaining extracts with potential antimicrobial activity. The aim of this study was to determine the antagonistic potential of whole pseudo-fruit and flesh extracts of three *Rosa* sp. varieties against *Staphylococcus* spp. bacteria isolated as food contaminants.

## 2. Results and Discussion

Table 1 shows the polyphenolic composition of freeze-dried preparations obtained from the pseudo-fruit and edible part of the flesh (rose flesh) of *Rosa rugosa*, *Rosa canina*, and *Rosa pomifera* Karpatia. The tested preparations differed significantly in their polyphenol composition. The sum of polyphenols ranged from 28,862 to 35,358 mg/100 g of lyophilisate. The main groups of polyphenols found in the preparations were flavanols and ellagitannins. The preparations obtained from *Rosa rugosa* differed significantly in the content of ellagitanins, being nearly 5–8 times higher than in the other preparations. When comparing the fleshy part and the whole fruit, it was found that irrespective of the variety, the ellagitannin content in the fleshy part was always lower. A reverse relationship was observed for flavanols: their contents were higher in the extracts obtained from the edible parts of the flesh. The highest content of these compounds was noted in extracts from *Rosa canina*. There were also significant differences in the content of flavonols. Preparations from *Rosa rugosa* were characterised by a lower content of these compounds.

Table 2 shows the effect of *Rosa* sp. extracts on the growth of bacteria of the genus *Staphylococcus* isolated from food. All of the tested extracts inhibited the growth of staphylococci at a concentration of 500 mg/mL. *Rosa rugosa* fruit extract showed the strongest antimicrobial properties among the studied extracts. For all the strains, the growth inhibition had a diameter of 20.3–29.0 mm. Moreover, six out of the seven tested strains showed the highest inhibition with the use of this extract. The exception was *Staphylococcus capitis* KR6, for which the largest zone of growth inhibition was observed after the use of *Rosa pomifera* Karpatia whole-fruit extract (20.7 mm). It is worth noting, however, that this is not a statistically significant difference compared to the *Rosa rugosa* whole-fruit extract. Thus, whole-fruit extracts of the cultivars *Rosa pomifera* Karpatia and *Rosa rugosa* are characterised by a similar antagonistic potential against *Staphylococcus capitis* KR6. For the reference strain *Staphylococcus aureus* ATCC 25923, the extracts from the flesh of all tested rose species demonstrated similar antibacterial properties. The zone of inhibition of growth of the reference strain was comparable for all extracts (13.0–13.3 mm). However, in the case of the reference strain *Staphylococcus epidermidis* DSMZ 3270, the highest antibacterial potential was observed for the *Rosa rugosa* whole-fruit extract. The strains *Staphylococcus epidermidis* A5 and *Staphylococcus haemolyticus* M6 turned out to be the most resistant to *Rosa rugosa* flesh extracts, while *Staphylococcus xylosus* M5 and *Staphylococcus capitis* KR6 displayed resistance to *Rosa canina* flesh extracts. The *Staphylococcus warneri* KR2A strain is the most resistant to the action of *Rosa pomifera* Karpatia flesh extract. For all of the studied strains, it was observed that rose-flesh extracts have weaker antibacterial potential against *Stapylococcus* spp. strains than extracts prepared from whole fruits.

Table 2 presents the results which prove the high antibacterial potential of extracts from three species of *Rosa* spp. against the tested strains of *Staphylococcus* spp. which contaminate food. The interaction between the microorganism and compounds contained in the tested extracts is always specific and certainly depends on many factors, such as the origin of the tested strains or the variety of *Rosa* spp. fruit. An important factor in these interactions is the part of the fruit from which the extracts were obtained.

Table 3 presents the MIC value for the growth of *Staphylococcus* species. The MIC of rose extracts was in the range of 3.125–500 mg/mL and was strictly dependent on the bacterial species, the species of the rose, and the part of the fruit from which the extract was obtained. Lower MIC values were noted when whole-fruit extracts were used in the tests. For *Rosa canina* extracts, the MIC value was 3.125–500 mg/mL. In the case of whole-fruit extracts, the MIC was lower and ranged from 3.125 to 200 mg/mL, whilst for flesh extracts, it ranged from 200 to 500 mg/mL. A similar trend was observed in the case of *Rosa rugosa* extracts (MIC range: 3.125–500 mg/mL). Whole-fruit extracts inhibited the growth of *Staphylococcus* bacteria at concentrations of 3.125–100 mg/mL, while *Rosa rugosa* flesh extracts did so at concentrations of 12.5–500 mg/mL. In the case of *Rosa pomifera* Karpatia, the MIC value was 3.125–500 mg/mL, and it was also significantly lower in the case of whole-fruit extracts (3.125–300 mg/mL) than in the case of flesh extracts (50–500 mg/mL).

When analysing the lowest concentration of extracts from whole fruits and rose flesh which can inhibit the growth of the studied species of *Staphylococcus* bacteria, it can be seen that in the case of the reference strains, the lowest MIC values were achieved with the *Rosa rugosa* whole-fruit extract (3.125 mg/mL for *Staphylococcus aureus* ATCC 25923; 25 mg/mL for *Staphylococcus epidermidis* DSMZ 3270). The reference strains were more resistant to the action of rose-flesh extracts than those of the whole fruit. The MIC value of flesh extracts was as high as 500 mg/mL (*Staphylococcus aureus* ATCC 25923—*Rosa rugosa* flesh extract and *Rosa pomifera* Karpatia; *Staphylococcus epidermidis* DSMZ 3270—*Rosa canina*). In the case of strains isolated from radish sprouts, the lowest MIC value was obtained using the *Rosa rugosa* whole-fruit extract (12.5 mg/mL for *Staphylococcus capitis* KR6; 6.25 mg/mL for *Staphylococcus warneri* KR2A), whilst the highest MIC values were found for the *Rosa pomifera* Karpatia flesh extract (300 mg/mL for *Staphylococcus capitis* KR6; 500 mg/mL for *Staphylococcus warneri* KR2A). In the case of *Staphylococcus epidermidis* A5, the lowest MIC value was obtained for the whole-fruit extracts of *Rosa canina* and *Rosa polfera* Karpatia (3.125 mg/mL), and the highest was for the flesh extract of *Rosa rugosa* (400 mg/mL). For the strain *Staphylococcus xylosus* M5, the lowest MIC value was found for the *Rosa rugosa* whole-fruit extract (6.25 mg/mL), and the highest was for the *Rosa canina* flesh extract (300 mg/mL). *Rosa canina* and *Rosa rugosa* flesh extracts also showed the weakest antimicrobial properties against *Staphylococcus haemolyticus* M6 (500 mg/mL), though this strain was unable to grow at 50 mg/mL of *Rosa canina* whole-fruit extract (the lowest MIC value for this strain). To summarise, the most sensitive strains to the tested *Rosa* sp. fruit and flesh extracts were *Staphylococcus epidermidis* A5, *Staphylococcus xylosus* M5, and *Staphylococcus capitis* KR6.

Whole-fruit extracts have stronger antimicrobial properties than rose hips extracts. Whole-fruit extracts of *Rosa canina* fruit preparations displayed 2.5–96 times greater antagonistic activity. For *Rosa rugosa* and *Rosa pomifera* Karpatia, these values were 1–160 and 2–32, respectively. Research on the antimicrobial effect of *Rosa rugosa* fruit extracts was also conducted by Cendrowski et al. [35]. Their results confirm the inhibitory effect of the extracts against *Bacillus cereus*, *Staphylococcus aureus*, *Staphylococcus epidermidis*, *Listeria innocua*, *Escherichia coli*, and *Salmonella enterica*; they reported MICs for *Staphylococcus aureus* and *Staphylococcus epidermidis* of 16–64 mg/mL and 16–32 mg/mL, respectively, depending on the method of extraction. The antimicrobial effect of *Rosa canina* hip was presented by Ghendov-Moșanu et al. [36], who noted that the shredded fruit showed a strong inhibitory effect on *Staphylococcus aureus*.

Table 4 and Table 5 show the correlation between the main groups of polyphenols in the extracts and their inhibition of bacterial growth. In the case of pseudo-fruit extracts, the inhibitory effect on bacterial growth positively correlated with the content of ellagitannins, and this effect was observed for almost all the tested strains, except for *Staphylococcus capitis* KR6. However, in the case of the flesh extracts, a strong positive correlation with ellagitannins was observed only for *Staphylococcus epidermidis* DSMZ 3270 and *Staphylococcus xylosus* M5. Additionally, it was found that the presence of flavanols in the tested extracts did not have a significant growth inhibition effect on the tested strains. In some cases, even a strong negative correlation was observed, which indicates that these compounds may stimulate the growth of microorganisms. The inhibitory effect of individual ellagitannins on *S. aureus* was presented in studies by Puljula et al. [37]; among the tested compounds, salicarinin A and rugosin D completely inhibited the growth of *S. aureus*. Effective inhibition has also been shown by casuarictin, tellimagrandins I and II, pentagalloylglucose, stachyurin, casuarinin, vescalagin, castalagin, rugosin E, sanguiin H-6, and lambertianin C. The above-mentioned authors reported that *S. aureus* is the species most sensitive to tannins. Such results were also obtained by Puupponen-Pimia et al. [38], who showed that ellagitannin-rich blueberries and berry extracts inhibited the growth of *S. aureus*.

## 3. Materials and Methods

### 3.1. Microorganisms

The biological material in this study consisted of seven strains of bacteria from the genus *Staphylococcus*. Two strains—*Staphylococcus aureus* ATCC 25923 and *Staphylococcus epidermidis* DSMZ 3270—were used as reference strains. The other five strains were food-derived isolates. The species affiliation of the isolates was confirmed by molecular methods based on sequence analysis of the 16S ribosomal RNA gene. The sequences of the strains have been deposited in the GenBank biotechnology database (US National Center for Biotechnology Information, Bethesda, MD, USA). The species affiliation and the sequence access numbers of the tested strains are presented in Table 6. All strains (stored in MAST CRYOBANK; Mast Group Ltd., Merseyside, UK), were activated by transfer to a fresh nutrient medium (Merck, Darmstadt, Germany) and culturing at 37 °C for 24 h.

### 3.2. Antagonist Activity and Minimum Inhibitory Concentration for Extracts from Rosa Spp.

From a 24 h culture of *Staphylococcus* bacteria with a density of 10^8^ CFU/mL, 1 mL of the suspension was taken and then spread onto a sterile petri dish and covered with cooled nutrient agar (filled to the upper limit of the plate height, using approximately 20 mL of medium). After the agar had solidified, six wells were cut out of the plate with a sterile cork bore (the cut disks were removed with a sterile needle). The holes were filled with 100 µl of appropriately diluted *Rosa* sp. extracts (previously prepared by dissolving the lyophilised extracts in 5% (*v/v*) DMSO (Sigma-Aldrich, St. Louis, MO, USA) (range of extract concentration: 0.006–500 mg/mL). The negative control was 5% DMSO. The plates were then placed in an incubator (37 °C) for 18–24 h. After this time, the zones of inhibition of growth of the *Staphylococcus* bacteria were measured. The result is given in mm. Total antagonistic activity was determined for *Rosa* sp. extracts at a concentration of 500 mg/mL. The study was performed in three independent replications. The minimum inhibitory concentration (MIC) is the lowest concentration (in the range of 0.006–500 mg/mL) at which a zone of inhibition of bacterial growth was observed.

### 3.3. Obtaining the Extracts

**Figure 1:**
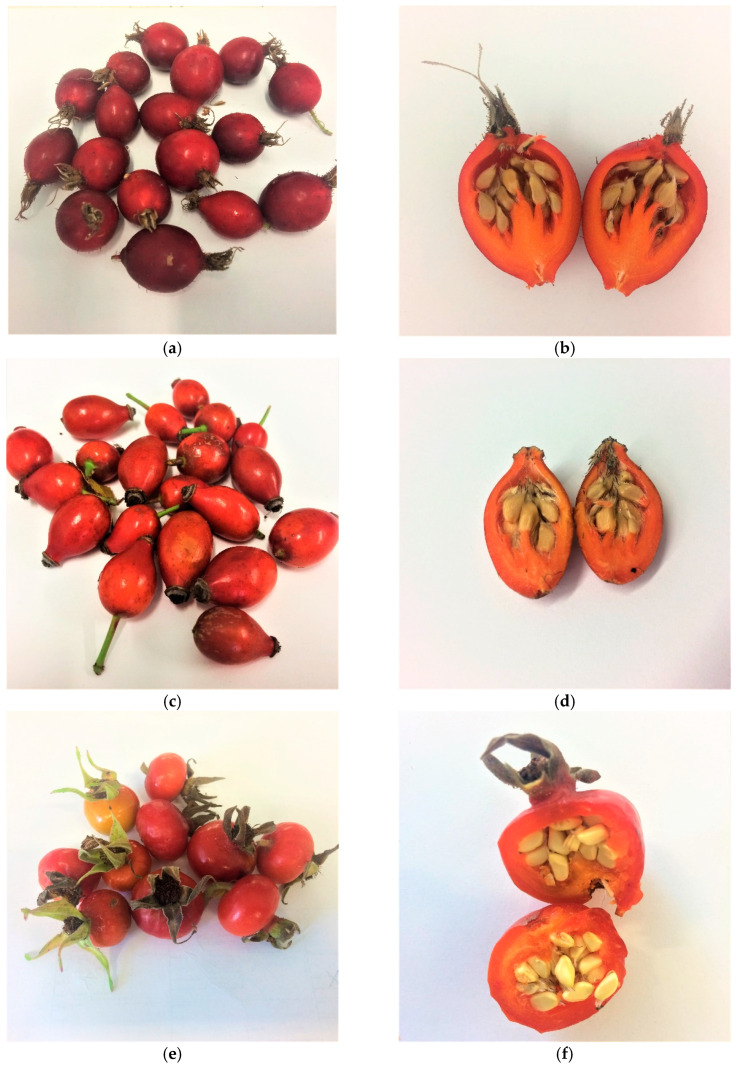
Pseudo-fruits of the studied varieties of rose species: *Rosa pomifera* Karpatia (**a**) whole peseudo-fruit and (**b**) pseudo-fruit cross section, *Rosa canina* (**c**) whole pseudo-fruit and (**d**) pseudo-fruit cross section, *Rosa rugosa* (**e**) whole pseudo-fruit and (**f**) pseudo-fruit cross section.

The material was the pseudo-fruits of *Rosa canina*, *Rosa pomifera* Karpatia, and *Rosa rugosa* obtained from the National Institute of Horticultural Research in Skierniewice, Poland (Figure 1). The polyphenols were extracted from the fleshy part and the whole pseudo-fruit for all rose varieties. Before extraction, the material was ground with liquid nitrogen in an NMK 110 crusher (SPOMASZ-Nakło, Nakło nad Notecią, Poland). The ground material was covered with 60% acetone acidified with 0.05% HCOOH (pH: 3.5) at a ratio of 1:5, and extraction was performed on a DOS-10L orbital shaker (ELMI SIA, Riga, Latvia) at 135 rpm/min. Extraction by shaking was carried out for 8 h, whilst the next step was static (total extraction time: 24 h). At a later stage, Extract I was decanted and the material was re-extracted in the same manner to obtain Extract II. The combined extracts from steps I and II were concentrated on a Heidolph automated 24/7 continuous evaporator. The concentrated extracts were purified in an XAD 1600 column (dimensions: 50 × 7 cm) filled with a bed up to a height of 34 cm. The concentrated crude extracts were applied to the column, then the column was washed with water and the adsorbed polyphenols were eluted with 10% and 60% methanol acidified with 0.01% HCOOH. The methanol fractions were combined and concentrated on a Heidolph 24/7 automatic evaporator. Next, the extract was lyophilised in a Christ Alpha 1–2 LD plus laboratory freeze dryer.

The resulting extracts were tested for polyphenols.

### 3.4. HPLC-FD Measurement of Flavanols and Procyanidins

The content of procyanidins was determined using the proanthocyanidin degradation method in an acidic environment using an excess of phloroglucinol, according to Kennedy et al. [39] with modifications. In 2 mL polypropylene test tubes, 10 ± 0.01 mg of the lyophilised material was weighed, and then 0.8 mL of a methanolic solution containing ascorbic acid and phloroglucinol at concentrations of 15 and 75 g/L, respectively, was added. The phloroglucinolysis reaction was started by adding 0.4 mL of 0.2 M hydrochloric acid in anhydrous methanol; incubation was performed on a Grant-bio PHMT shaker (Grant, Cambridgeshire, England) at 1200 RPM for 30 min at 50 °C. After incubation, the samples were immediately cooled in an ice bath for 3 min, and then 0.6 mL of a 40 mM sodium acetate aqueous solution was added. The samples were centrifuged for 5 min at 14,000 rpm and then diluted 5–10 times with the 40 mM sodium acetate solution. The diluted samples were then subjected to chromatographic analysis. The content of free catechins was determined from the solutions obtained after dissolving 40 mg of the lyophilised extract in 10 mL of 70% methanol. The content of phloroglucinolysis products and free catechins was determined using a Shimadzu chromatograph (Shimadzu, Tokyo, Japan) equipped with an RF-10XL fluorescence detector (Shimadzu, Tokyo, Japan) on a Gemini 5 µC18 110A column (250 × 4.6 mm; 5 µm). The mobile phases consisted of phase A—2.5% aqueous acetic acid solution (*v/v*)—and phase B—80% acetonitrile in water (*v/v*). The column temperature was 30 °C, the flow rate was 1 mL/min, the injection volume was 20 µl, and the separation time was 50 min. The gradient in phase B was applied as follows: 0–10 min, 4–7%; 10–27 min, 7–30%; 27–30 min, 30–70%; 30–34 min, 70%; 34–35 min, 70–4%; and 35–50 min, 4%.

The compounds were identified by comparing the retention times of the adducts: (−)-epicatechin-phloroglucinol adducts, (+)-catechin-phloroglucinol adducts, and (−)-epicatechin and (+)-catechin. The quantitative analysis of flavan-3-ols, i.e., adducts and released and free (−)-epicatechin and (+)-catechin, was performed using chromatograms recorded by the fluorescence detector with the excitation wavelength set to 278 nm and the emission wavelength set to 360 nm. For the calculations of the released and free (−)-epicatechin and (+)-catechin, calibration curves determined for (+)-catechin and (−)-epicatechin were used; (−)-epicatechin-phloroglucinol adducts were used for the calculation of adducts.

### 3.5. Qualitative and Quantitative Measurement of Selected Polyphenols by UHPLC-DAD-MS

Prior to analysis, the extracts were weighed out to an amount of approximately 80 mg and dissolved in 10 mL of 70% methanol. The solutions obtained in this way were diluted 1:1 with phase A, centrifuged at 12,000× *g*, transferred to vials, and analysed by LC-MS chromatography.

The qualitative and quantitative analyses of ellagitannins, ellagic acid, and flavonols were performed on a Dionex Ultimate 300 UHPLC chromatograph with a diode-array detector (DAD) coupled to a Q Exactive Orbitrap mass spectrometer (MS; Thermo Fisher Scientific, Waltham, MA, USA) on a Luna Omega 1.6 μm XB-C18 100 A column (150 × 2.1 mm) with a precolumn (Phenomenex, Torrance, CA, USA) using Thermo-Xcalibur 3.063 software (Thermo Fisher Scientific). The mobile phases were phase A—a 0.5% formic acid solution—and phase B—acetonitrile:methanol:water:formic acid (63:20:16.5:0.5) (*v/v/v/v*). The flow of the mobile phase was 0.4 mL/min, the injection volume was 5 µl, and the temperature was 40 °C. The gradient separation for phase B lasted 35 min and proceeded as follows: 0–2 min, 5%; 2–12 min, 5–28%; 12–20 min, 28–73%; 20–25 min, 73%; 25–27 min, 73–5%; 27–30 min, 5%. In order to quantify the compounds, the following standards were used: agrimoniin and bis-HHDP-glucose, pedunculagin (obtained from the Institute of Food Technology and Analysis, Łódź, Poland), quercetin-3-glucoside, tiliroside, kaempferol 3-glucoside, ellagic acid, quercetin 3-d-galactoside, quercetin, and kaempferol (Extrasynthese, France).

The qualitative determination was based on data obtained from the MS and the DAD. Quantification was performed using a DAD based on calibration curves prepared for individual standards. Two wavelengths were used for detection: 250 and 360 nm. The MS system coupled to the UHPLC was equipped with an H-ESI probe used in negative mode. The source parameters were as follows: vaporiser temperature, 480 °C; ion spray voltage, 3.8 kV; capillary temperature, 350 °C; and sheath gas and auxiliary gas flow rates of 65 and 10 units, respectively. The detector was operated in either full MS or full MS/dd-MS2 scanning modes. In the full MS mode, a scan range of 150–2000 *m/z* was used. To generate MS2 data, the full MS/dd-MS2 scanning mode was applied. The collision energy used to generate the MS2 spectra was set to 20.

### 3.6. Validation Parameters of HPLC Methods

#### 3.6.1. Linearity

Standard calibration curves were prepared using the following standards: agrimoniin and bis-HHDP-glucose pedunculagin (obtained from the Institute of Food Technology and Analysis), quercetin-3-glucoside, tiliroside, kaempferol 3-glucoside, ellagic acid, quercetin 3-d-galactoside, quercetin, kaempferol, (−)-epicatechin-phloroglucinol adduct (after phloroglucinolysis of procyanidin B2 standard) (Extrasynthese, France), (−)-epicatechin and (+)-catechin (Sigma, Steinheim, Germany). Standard stock solutions were diluted to appropriate concentrations for the plotting of calibration curves. The linearity was obtained by plotting the peak areas versus the corresponding concentrations (mg/L) of each analyte. Table 7 shows the equations of the calibration curves and the correlation coefficients (R^2^) of the standards used.

#### 3.6.2. Limit of Detection (LOD) and Limit of Quantification (LOQ)

For HPLC method used for ellagitannins and flavonols, the LOD and LOQ were determined based on the S/N (signal-to-noise) ratio. After appropriate dilution, the standard stock solutions were injected into chromatographs and the S/N ratio was recorded for each concentration of the test compound. LODs and LOQs were determined at an S/N ratio of approximately 5 and 10, respectively. In the case of the HPLC method used for flavanol and procyanidin, the LOD and LOQ were determined based on the standard deviation of response and slope.

### 3.7. Statistics

The data regarding the polyphenol content in the extracts and the antagonistic activity of the extracts were statistically analysed using Statistica 12 software (StatSoft, Tulsa, OK, USA), and analysis of variance (ANOVA) was used with Tukey’s and Duncan’s post hoc tests (*p* ≤ 0.05). Additionally, the influence of selected groups of polyphenols on their antagonistic activity against the tested strains was assessed using correlation analysis.

## 4. Conclusions

The results show the possibility of using the ellagitannin-rich extracts from rose (*Rosa canina*, *Rosa pomifera* Karpatia, and *Rosa rugosa*) pseudo-fruits to effectively limit the growth of bacteria from the genus *Staphylococcus*. *Rosa rugosa* fruit extract showed the strongest antimicrobial properties. For all of the studied strains, whole-fruit extracts had stronger antibacterial potential against staphylococci than extracts prepared from the flesh of the tested rose species. In the case of *Rosa rugosa*, whole-fruit extracts inhibited the growth of *Staphylococcus* bacteria at concentrations of 3.125–100 mg/mL (12.5–500 mg/mL for flesh extracts). The most sensitive strains to the tested *Rosa* sp. extracts were *Staphylococcus epidermidis* A5, *Staphylococcus xylosus* M5, and *Staphylococcus capitis* KR6. For almost all the tested strains (except *Staphylococcus capitis* KR6), the inhibitory effect on bacterial growth positively correlated with the content of ellagitannins. It should be emphasised that the results presented herein follow the current trend of minimising the use of chemical preservatives in food.

## Figures and Tables

**Table 1:** Polyphenolic composition of preparations of lyophilised extracts.

Preparation	Ellagitannins (MS Data (*m/z*) ≤ (1256)^−^	Ellagitannins (*m/z*) > (1256)^−^	Dominant Ellagitannin (*m/z*) (934)^−2^	Sum of Ellagitaninns	Sum of Ellagitaninns and Ellagic Acid	Flavonols	Flavanols	Procyanidins	Total Polyphenols
	[mg/100g]								
RRC	4425.2 ± 10.9 ^f^	6565.7 ± 95.4 ^e^	3515.9 ± 55.1 ^e^	11588.7 ± 98.2 ^f^	12226.6 ± 97.1 ^f^	213.4 ± 2.7 ^a^	20971.9 ± 737.4 ^a^	20564.6 ± 737.3 ^a^	33411.9 ± 835.2 ^bc^
RRM	2493.8 ± 31.6 ^e^	4557.6 ± 35.7 ^d^	2010.5 ± 21.1 ^d^	7051.5 ± 49.7 ^e^	7249.4 ± 50.6 ^e^	217.6 ± 3.2 ^a^	24690.4 ± 1221.1 ^b^	24656.9 ± 1222.2 ^b^	32157.4 ± 1266.3 ^b^
RCC	1027.6 ± 12.7 ^b^	636.4 ± 8.6 ^ab^	473.9 ± 9.2 ^b^	1664.0 ± 11.7 ^b^	1725.9 ± 12.6 ^b^	505.7 ± 6.8 ^d^	30159.4 ± 2641.6 ^c^	29395.6 ± 2633.9 ^c^	32391.1 ± 2646.1 ^bc^
RCM	416.2 ± 4.7 ^a^	693.8 ± 6.3 ^b^	491.3 ± 14.9 ^b^	1110.1 ± 1.8 ^a^	1146.6 ± 1.4 ^a^	384.1 ± 4.1 ^b^	33828.5 ± 1792.0 ^d^	33767.2 ± 1790.2 ^d^	35358.8 ± 1789.6 ^c^
RKC	1496.2 ± 9.2 ^d^	1106.1 ± 10.8 ^c^	878.6 ± 13.0 ^c^	2602.3 ± 19.9 ^d^	2715.3 ± 20.9 ^d^	506.8 ± 2.4 ^d^	25806.1 ± 1654.4 ^b^	25389.4 ± 1567.2 ^b^	29028.2 ± 1648.1 ^a^
RKM	1196.2 ± 7.0 ^c^	599.3 ± 4.6 ^a^	413.8 ± 1.1 ^a^	1795.5 ± 10.8 ^c^	1834.4 ± 11.4 ^c^	443.8 ± 6.2 ^c^	26584.0 ± 769.6 ^b^	26528.2 ± 769.9 ^b^	28862.1 ± 772.1 ^a^

RR—*Rosa rugosa*; RC—*Rosa canina*; RK—*Rosa pomifera* Karpatia; C—pseudo-fruit; M—flesh. The mean values (±standard deviation) marked in the columns with the same letter do not differ statistically significantly at the level of *p* = 0.05. The results were subjected to statistical analysis using one-way ANOVA and Duncan’s test.

**Table 2:** Antagonistic activity of *Rosa* spp. extracts (at a concentration of 500 mg/mL), obtained from whole fruits and the flesh alone, against *Staphylococcus* spp. strains.

	Growth Inhibition Zone [mm]					
	*Rosa canina*		*Rosa rugosa*		*Rosa pomifera* Karpatia	
	Whole Fruit	Flesh	Whole Fruit	Flesh	Whole Fruit	Flesh
ATCC 25923	20.7 ± 0.6 ^a^*	13.3 ± 0.6 ^A#^	23.3 ± 0.6 ^b^*	13.0 ± 0.0 ^A#^	20.7 ± 1.5 ^a^*	13.0 ± 0.0 ^A#^
DSMZ 3270	15.7 ± 0.6 ^a^*	12.0 ± 0.5 ^A#^	23.3 ± 2.1 ^b^*	14.3 ± 1.2 ^B#^	18.3 ± 0.6 ^a^*	13.0 ± 0.0 ^AB#^
A5	24.3 ± 0.6 ^a^*	15.7 ± 1.5 ^A#^	29.0 ± 1.1 ^b^*	13.7 ± 0.6 ^A#^	22.8 ± 0.6 ^c^*	19.0 ± 1.0 ^B#^
M5	20.7 ± 0.6 ^a^*	17.7 ± 0.6 ^A#^	24.3± 0.6 ^b^*	23.0 ± 0.0 ^B#^	23.0 ± 0.0 ^c^*	19.3 ± 0.6 ^C#^
M6	18.3 ± 0.1 ^a^*	12.0 ± 0.0 ^A#^	20.3 ± 0.6 ^b^*	11.7 ± 0.6 ^A#^	19.0 ± 1.1 ^ab^*	13.3 ± 0.6 ^B#^
KR6	1.7 ± 0.6 ^a^*	17.0 ± 0.0 ^A#^	20.3 ± 0.6 ^b^*	17.5 ± 1.2 ^A#^	20.7 ± 0.6 ^b^*	17.0 ± 1.7 ^A#^
KR2A	18.0 ± 0.0 ^a^*	15.5 ± 1.0 ^A#^	21.7 ± 0.6 ^b^*	14.7 ± 0.6 ^AB#^	19.7 ± 1.5 ^ab^*	13.0 ± 0.0 ^B#^

^a, b, c^—statistical differences (ANOVA, Tukey’s post-hoc test (*p* ≤ 0.05)) between whole fruit extracts within one test strain; ^A, B, C^—statistical differences (ANOVA, Tukey’s post-hoc test (*p* ≤ 0.05)) between flesh extracts within one test strain. *, ^#^—statistical differences (ANOVA, Tukey’s post-hoc test (*p* ≤ 0.05)) between extracts (whole fruits—flesh alone) obtained from the same *Rosa* sp. species within one test strain.

**Table 3:** Minimum inhibitory concentration of whole fruit and flesh extracts from *Rosa* sp. for the tested *Staphylococcus* spp. strains.

	MIC [mg/mL]					
	*Rosa canina*		*Rosa rugosa*		*Rosa pomifera* Karpatia	
	Whole Fruit	Flesh	Whole Fruit	Flesh	Whole Fruit	Flesh
ATCC 25923	12.5	300	3.125	500	25	500
DSMZ 3270	200	500	25	400	300	400
A5	3.125	300	6.25	400	3.125	100
M5	50	300	6.25	12.5	12.5	50
M6	50	500	100	500	100	400
KR6	25	200	12.5	50	25	300
KR2A	25	300	6.25	400	100	500

**Table 4:** Correlation between the ability to inhibit bacterial growth (*Staphylococcus* spp.) and the main groups of polyphenols present in the pseudo-fruit extracts of selected rose cultivars.

Variable	Correlation (*p* < 0.050) Microorganism						
	ATCC 25923	DSMZ 3270	A5	M5	M6	KR6	KR2A
Sum of ellagitannins	0.837 *	0.913 *	0.905 *	0.805 *	0.814 *	0.388	0.825 *
Flavanols	−0.527	−0.921 *	−0.623	−0.890 *	−0.746 *	−0.593	−0.741 *
Procyanidins	−0.526	−0.921 *	−0.634	−0.882 *	−0.740 *	−0.576	−0.734 *
Total polyphenols	0.712 *	0.198	0.688 *	0.044	0.301	−0.252	0.330

*—there is a correlation between polyphenolic compounds and microorganism.

**Table 5:** Correlation between the ability to inhibit bacterial growth (*Staphylococcus* spp.) and the main groups of polyphenols present in the extracts from the flesh of selected rose cultivars.

Variable	Correlation (*p* < 0.050) Microorganism						
	ATCC 25923	DSMZ 3270	A5	M5	M6	KR6	KR2A
Sum of ellag-itannins	−0.294	0.824 *	−0.662	0.965 *	−0.504	0.299	0.218
Flavanols	0.369	−0.699 *	0.110	−0.829 *	−0.163	−0.325	0.350
Procyanidins	0.369	−0.698 *	0.110	−0.838 *	−0.165	−0.325	−0.351
Total poly-phenols	0.251	−0.234	−0.447	−0.304	−0.689 *	−0.188	0.691 *

*—there is a correlation between polyphenolic compounds and microorganism.

**Table 6:** Species affiliation of the studied strains of the genus *Staphylococus.*

Species	Nucleotide Sequence Number	Isolate Symbol	Origin
***Staphylococcus epidermidis***	MW 040699	A5	Dietary supplement—acai berries extract
***Staphylococcus xylosus***	MW 776359	M5	Fresh milk
***Staphylococcus haemolyticus***	MW 776358	M6	Fresh milk
***Staphylococcus capitis***	MW 776357	KR6	Radish sprouts
***Staphylococcus warneri***	MW 776360	KR2A	Radish sprouts

**Table 7:** Validation parameters of the methods used for polyphenol determination.

Compound	Calibration Curves	R^2^	LOD (mg/L)	LOQ (mg/L)
Pedunculagin bis-HHDP-glucose	y = 6263.4x − 2220.5	R^2^ = 0.9989	0.29	0.59
Agrimoniin	y = 11296x − 1056	R^2^ = 0.9999	0.16	0.32
Quercetin-3-glucoside	y = 15513x + 2283.2	R^2^ = 0.9996	0.11	0.22
Quercetin 3-d-galactoside	y = 19134x + 1932.3	R^2^ = 0.9999	0.09	0.18
Tiliroside	y = 11305x + 2734.3	R^2^ = 0.9998	0.15	0.30
kaempferol 3-glucoside	y = 15287x − 166.39	R^2^ = 0.9997	0.11	0.22
Quercetin	y = 24852x − 1257.3	R^2^ = 0.9992	0.07	0.14
Kaempferol	y = 18344x − 2565.7	R^2^ = 0.9999	0.09	0.18
Elllagic acid	y = 17521x − 816.99	R^2^ = 0.9998	0.10	0.20
(−)-epicatechin-phloroglucnol adduct	y = 75639x + 43737	R^2^ = 0.9974	0.34	0.68
(−)-epicatechin	y= 98857x − 1666.1	R^2^ = 0.9999	0.03	0.06
(+)-catechin	y = 99608x + 40.24	R^2^ = 0.9999	0.04	0.08

R^2^—correlation coefficient; LOD—limit of detection; LOQ—limit of quantification.

## Data Availability

The data presented in this study are available on request from the corresponding author.

## Sample Availability

Samples of the compounds are not available from the authors.
